# In silico optimization of aligned fiber electrodes for dielectric elastomer actuators

**DOI:** 10.1038/s41598-024-54931-y

**Published:** 2024-02-27

**Authors:** Mohammadreza Firoozan, Majid Baniassadi, Mostafa Baghani, Alex Chortos

**Affiliations:** 1 School of Mechanical Engineering, Purdue University, West Lafayette, USA; 2 School of Mechanical Engineering, University of Tehran, Tehran, Iran

**Keywords:** Mechanical engineering, Applied physics, Polymers

## Abstract

Dielectric elastomer actuators (DEAs) exhibit fast actuation and high efficiencies, enabling applications in optics, wearable haptics, and insect-scale robotics. However, the non-uniformity and high sheet resistance of traditional soft electrodes based on nanomaterials limit the performance and operating frequency of the devices. In this work, we computationally investigate electrodes composed of arrays of stiff fiber electrodes. Aligning the fibers along one direction creates an electrode layer that exhibits zero stiffness in one direction and is predicted to possess high and uniform sheet resistance. A comprehensive parameter study of the fiber density and dielectric thickness reveals that the fiber density primary determines the electric field localization while the dielectric thickness primarily determines the unit cell stiffness. These trends identify an optimal condition for the actuation performance of the aligned electrode DEAs. This work demonstrates that deterministically designed electrodes composed of stiff materials could provide a new paradigm with the potential to surpass the performance of traditional soft planar electrodes.

## Introduction

New frontiers in application spaces for actuators, such as bio-integrated devices and distributed systems^1,2^, have motivated the development of polymer-based actuators^3–5^ that have advantages such as intrinsic compliance, size-independent performance, low cost, and compatibility with earth-abundant and biodegradable materials. Electrostatically-driven polymer actuators rely on electrostatic attraction between oppositely charged electrodes to deform a soft capacitor. This direct conversion of electrical to mechanical energy produces a fast and efficient actuation^3^ that make them suitable for applications such as advanced optics^6,7^, micro-robotics^11,12^, and human–machine interfaces^13–16^. There are two choices of dielectrics for electrostatic polymer actuators: (1) dielectric elastomer actuators (DEAs) use solid elastomers, while (2) electrohydraulic actuators^17^ employ a combination of flexible and liquid dielectric components. Electrohydraulic actuators provide higher actuation forces and variable geometries, while DEAs can scale to smaller sizes and possess several orders of magnitude higher actuation frequencies. This combination of features (scalability, compliance, and) therefore make DEAs preferrable for applications such as microrobots^12,11^, optical devices, pumps and valves^18,19^, and tools for studying biology^20^. Applications in human machine interfaces are motivated by their intrinsic compliance and compatibility with high-frequency operation. Despite promising demonstrations using lab-scale DEAs, their industry adoption has been limited by high operation voltages and challenges with reliable manufacturing and long-term operation.

At small strains, the actuation stress ($stress=\varepsilon {E}^{2}$) and actuation strain ($strain=\varepsilon {E}^{2}/Y$) are linearly related to the dielectric constant ($\varepsilon$) and quadratically related to the electric field ($E$)^21^. The actuation strain is also inversely related to the Young’s modulus (Y). Consequently, the maximum electric field at which the device fails is the most important factor determining the actuation metrics of a DEA. The operating voltage can be moderately decreased by increasing the dielectric constant (ε_r_), but decreasing the dielectric thickness can achieve larger reductions in voltage. Practical DEAs will therefore be enabled by developing device structures and fabrication processes that enable reliable breakdown fields with small dielectric thicknesses. The choice and design of electrodes have important impacts on these key parameters.

The breakdown field of a DEA is determined by a complex interplay between mechanical and electrical factors. The electrodes have a large impact on both the electrical and mechanical properties, making them critical to the device performance. Electrodes are electrically necessary to charge the DEA capacitor. However, electrode stiffness increases the effective value of *Y* and reduces the actuation performance^22^. Consequently, most DEAs have employed very soft electrodes such as conductive hydrogels^23,24^, carbon grease^21,25^, or elastomers filled with carbon black^26–28^. In applications where high forces are required, multiple dielectric layers are typically stacked together^13,15,29–31^. In these multilayer devices, electrodes are desired to have a small thickness, high conductivity, and low in-plane stiffness. Common electrodes include carbon black powder and submonolayers of carbon nanotubes^32–35^. However, these electrodes based on nanomaterials have several limitations, including degradation over time^36^ and a tradeoff between thickness and sheet resistance^37^.

The stochastic nature of the deposition of nanomaterials leads to nonuniform conductivity within an electrode sheet. As a result, high-frequency actuation of the devices can generate thermal hotspots that correlate with faster breakdown^38,39^. Most importantly, aggregates of particles or misaligned carbon nanotubes can cause premature breakdown. For example, aggregates of carbon black particles can act as defect sites that reduce the breakdown field by 50%^4^, resulting in a 75% reduction in the performance metrics^40^. Similarly, when used in a multilayer fabrication process consisting of transfer printing of carbon nanotube electrodes followed by spin-coating dielectric layers, carbon nanotubes can become dispersed in the dielectric^41^, reducing the breakdown field. Furthermore, the sharp points of nanomaterials such as carbon nanotubes can lead to electric field localizations that lower the lifetime and breakdown field. These properties of nanomaterials limit the thicknesses of dielectrics^22^. With a constant size of the defect, as the dielectric thickness becomes smaller, the size of the defect relative to the dielectric thickness rises, further exacerbating the negative effect of the defect on the performance^40,42–46^. Electrode structures are one of the key factors limiting further reductions in the operating voltage of DEAs.

**Figure 1:**
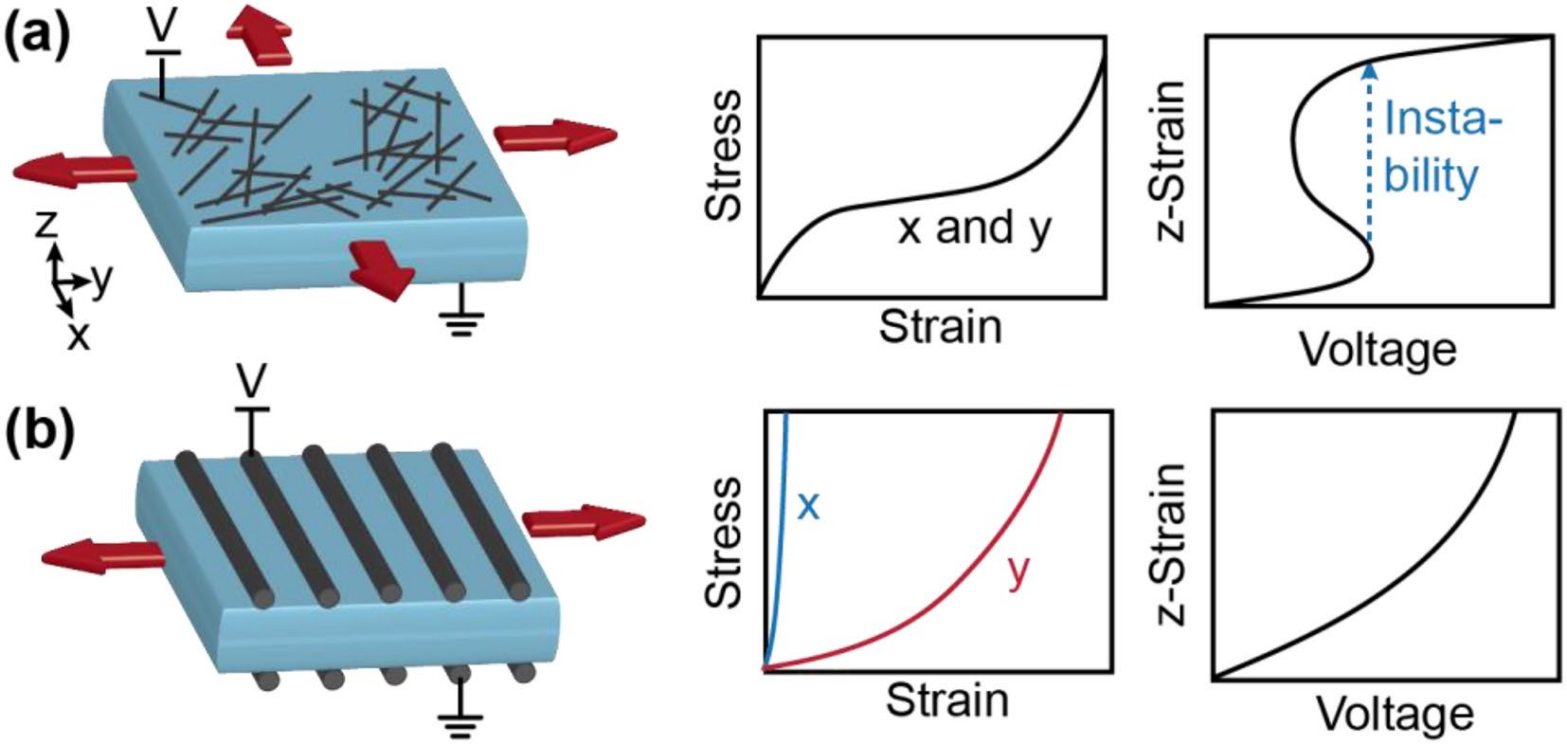
Conceptual comparison of electrodes based on nanomaterials and aligned fibers. (**a**) Biaxial actuation with traditional soft electrodes leads to electromechanical instability. (**b**) Uniaxial actuation in fiber-constrained devices suppresses electromechanical instability.

In addition to electrical failure due to the presence of defects, electromechanical instability has been a persistent challenge in the field of DEAs^47^. Electromechanical instability arises when the dielectric exhibits strain softening observed in most elastomers. Actuation of the device simultaneously reduces the thickness and the tangent modulus, resulting in a non-monotonic region of the strain as a function of voltage (Fig. 1a), causing simultaneous electrical and mechanical failure of the device. It can be prevented by using dielectric layers that have monotonically increasing tangent modulus as a function of strain, which may be accomplished through materials chemistry^47–53^, prestraining of the dielectric^54,55^, or geometric design. For example, adding stiffening fibers that constrain the actuation in one direction can delay or prevent electromechanical instability^56,57^ (Fig. 1b).

Geometric designs that introduce anisotropy can also be valuable for applications that benefit from linear actuation. In an isotropic DEA with soft electrodes, actuation results in a negative strain along the thickness direction and a corresponding positive strain along the radial direction. However, many applications only use the positive actuation in one direction (uniaxial). Consequently, several approaches have been developed to constrain the actuation along one direction, such as anisotropic elastomers based on aligned polymer chains^58,59^. Alternatively, device-level approaches include adding stiff mechanical constraints on the exterior of actuators^56,60,61^ or preparing anisotropic CNT electrodes^62,63^. These device structure approaches are advantageous because they can be used with any elastomer chemistry and do not require prestrain.

These device considerations—electrical breakdown, electromechanical stability, and anisotropy—are intimately related to the electrode design of DEAs. While most DEAs are engineered to have electrodes with very low modulus to minimize the effective Young’s modulus and maximize the actuation strain, some works aim to utilize stiff electrode materials with geometrically-engineered deformability. For example, serpentine electrodes provide low in-plane stiffness in two directions with very low sheet resistance^64^. Aligned electrodes of carbon nanotubes^62,63^ provide partially deterministic properties such as anisotropic stiffness in different directions but may still include defect-related drawbacks of nanomaterial electrodes. While these works have shown that the concept of stiff electrode materials is intriguing, they do not yet show improved performance metrics compared to conventional soft electrodes. Consequently, parametrized optimization studies are required to rationally design electrode structures.

In this work, we computationally explore the design of electrodes for multilayer DEAs in which the electrodes consist of aligned stiff fibers which we refer to as an aligned electrode DEA (AED). These types of electrodes are enabled by the increasing performance and reliability of new printing techniques such as direct ink write printing^65^, electrohydrodynamic printing, and inflight fiber printing^66^. This geometry has several advantages, including low sheet resistance, deterministic arrangements that minimize defects, and anisotropic mechanical properties that limit global instability. While the effect of stiff fibers on the actuation of DEAs has been analyzed previously^56^, these fibers were insulating dielectric materials, allowing the electric field in the dielectric to be assumed as uniform. When the fibers are conductive electrodes, the effect of nonuniform electric field distributions in the dielectric must be taken into account. We implement a dynamic nonlinear finite element model to analyze the actuation of DEAs as a function of the structural design of the stiff fiber electrodes.

## Results

### Simulation approach

Analytical models of anisotropic DEAs that are constrained in one direction predict that this anisotropy delays or prevents instability^56,57,67^. However, these models assume a constant electric field within the dielectric created by a uniform electrode. Fiber-based electrodes develop an electric field distribution within the dielectric that is nonuniform because of the discontinuous nature of the electrodes. Finite element (FEM) simulations enable accounting for these nonuniformities on the actuation performance. Within a simulation, the actuator is predicted to fail when any node in the simulation reaches the electrical breakdown field of the material. In a quasistatic simulation, the simulation will fail if the device reaches electromechanical instability^67–69^. In some cases, this instability point is reached before the electrical breakdown criterion is met. Dynamic models allow the simulation to proceed beyond the instability to reach the electrical failure condition (target local electrical field). Dynamic behavior has been implemented in the simulation of DEAs using several approaches, including the effect of inertia and the effect of viscoelasticity. Since the purpose of the dynamic simulation in this work is to simulate past instability for a quasistatic device, any method of implementing dynamic behavior is sufficient. Consequently, we use the inertia formulation due to its relative simplicity. Inertia formation can be implemented in a UEL in ABAQUS while the viscoelastic formulation is typically implemented as a UMAT. The mathematical formulation of the UEL is comprehensively provided in Supplementary Note 1.

To produce simulation results with intuitive numerical values, materials constants for common DEA materials were used: shear modulus (μ) of 80 kPa, relative dielectric constant (${\epsilon }_{r}$) of 3.2, and Poisson’s ratio of 0.495. The fibers are assigned a shear modulus of 80 MPa, which is 1000 times larger than the modulus of the dielectric.

Previous calculations on the use of rigid electrodes indicate that the largest actuation strain is expected for electrodes that have a low aspect ratio for the cross-section of the electrodes^70^. Consequently, we use electrodes that have a circular cross-section, which have the requisite low aspect ratio (aspect ratio of 1) and can be fabricated with printing or self-assembly methods. A rectangular representative volume element contains cylindrical electrodes extending in the y direction with one (2 $\times$ 0.5) high voltage electrode in the middle and one (4 $\times$ 0.25) low voltage electrode at the top and bottom. To simulate multilayer devices that are commonly used in high-energy DEAs, electrical and mechanical boundaries are set to be periodic in the x, y, and z directions.

**Figure 2:**
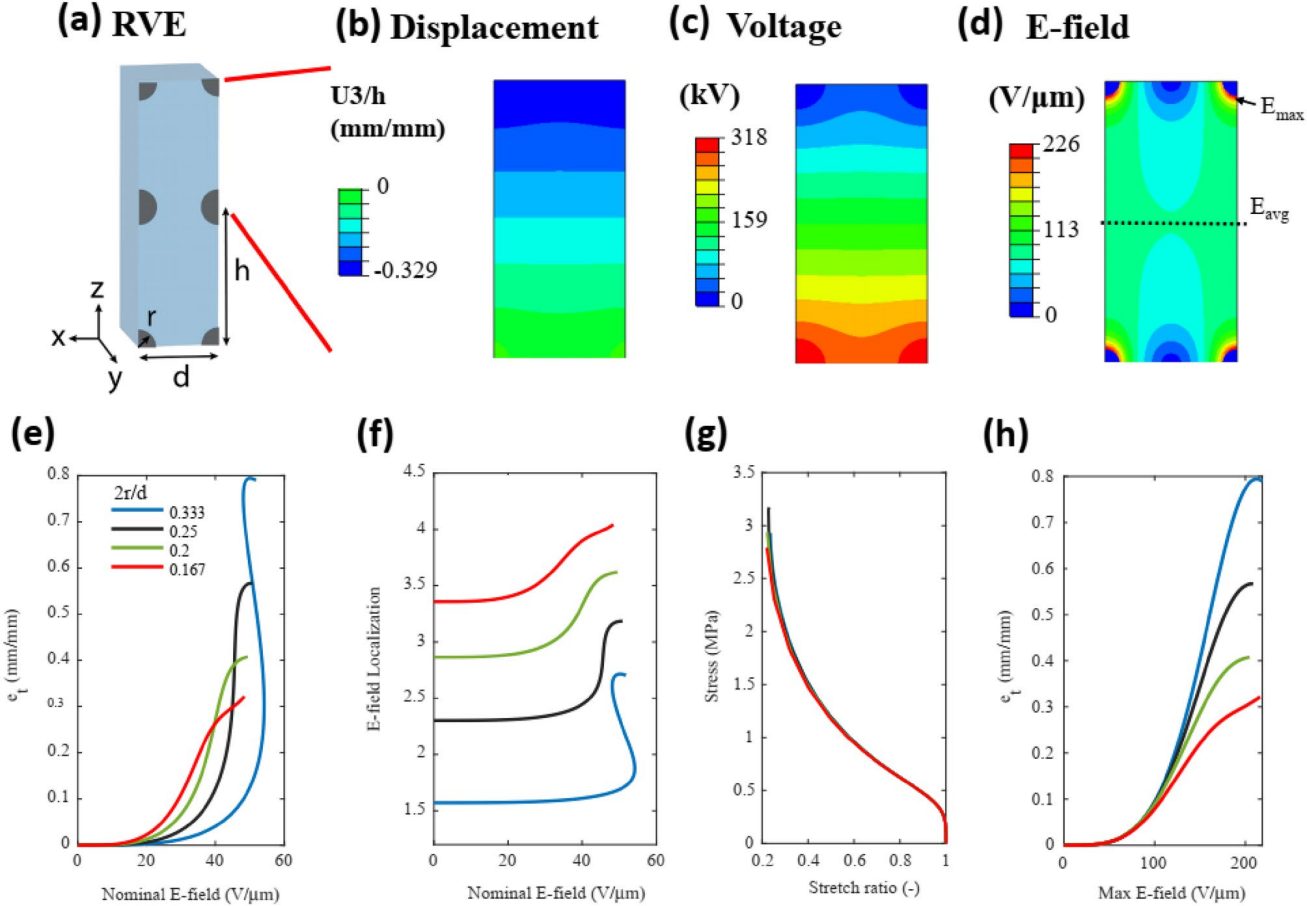
Actuation of a representative volume element with a fixed dielectric thickness (h/d = 5) and varying electrode density. (**a**) Schematic of the dimensions of the representative volume element. Images of finite element simulations at device failure: (**b**) displacement, (**c**) voltage distribution, and (**d**) electric field distribution. For select values of the electrode density (2r/d): (**e**) thickness actuation strain as a function of nominal E-field, (**f**) field localization (E_max_/E_bulk_), (**g**) compressive stress–strain behavior, and (**h**) thickness actuation strain as a function of the maximum E-field.

The key parameters that are varied in the simulation are the fiber radius (r), the distance between fibers (d), and the dielectric thickness (h) (Fig. 2a). These parameters are arranged into two non-dimensional values that relate to the physical device: the electrode density (2r/d) that represents the proportion of the RVE width (d) that is composed of electrodes, and the dielectric thickness (h/d). We first hold the dielectric thickness (h/d) constant and study the effect of the electrode density. In the second section, 2r/d was kept fixed and h/d values changed to indicate the effect of dielectric thickness relative to the fiber spacing (h/d).

### Effect of fiber density (2r/d)

Figure 2 shows the effect of the fiber density on the actuation strain and instability of the DEAs. As the actuation figure of merit, we use the engineering strain in the direction of the electric field: ${e}_{t} =\frac{\left|{U}_{3}\right|}{h}$, where ${U}_{3}$ is displacement along z-axis and h is the distance between low-voltage and high-voltage electrodes (Fig. 2a). The nominal E-field ($\widetilde{E}=V/{d}_{0})$ is calculated as the voltage divided by the initial dielectric thickness. Despite our unusual geometry that has discontinuous electrodes with non-zero thickness, we define the value of h as the dielectric thickness. The field localization is then quantified by comparing the largest value of the electric field in the dielectric (near the electrode) to the electric field in the middle of the dielectric, the average electric field $\left(Field \,Localization= \frac{{E}_{electrode}}{{E}_{avg}}\right)$.

Figure 2e shows the actuation strain of the device as a function of the nominal E-field. Plotting E-field localization based on nominal E-field (Fig. 2f) shows that higher fiber density leads to lower E-field localization due to the interaction between the electric fields surrounding the fibers. The electric field localization is constant for low electric fields (when there is little actuation), which we refer to as the low-field localization. As actuation proceeds, h decreases and d increases. This effectively decreases the value of h/d and decreases the value of 2r/d, which increases the E-field localization. This increase in the effective electric field within the dielectric causes positive feedback that can result in non-monotonic behavior in the actuation vs nominal E-field (Fig. 2e), which represents an instability. This type of instability due to positive feedback in the dielectric is also observed in dielectrics in which the average dielectric constant increases during actuation due to the interaction between inclusions with high dielectric constants^57^.

In addition to the electric field distribution, the mechanics of the unit cell influence the actuation. The stress–strain behavior of the unit cell is calculated by ramping a stress on the z-face of the unit cell and reporting the resulting displacement. Due to the stiff fibers, the strain in the direction of the fibers is very small. With a constant h/d value of 5, the compressive stress–strain behavior of the unit cell is nearly independent of the fiber spacing (Fig. 2g).

A dielectric material is generally considered to break down when any point within the dielectric reaches the breakdown field of the material. Consequently, we plot the actuation strain as a function of the maximum electric field at any point in the dielectric (Fig. 2h). At small actuations (< 0.1 mm/mm), the actuation strain vs max E-field for different fiber densities are very similar. This is somewhat non-obvious because reducing the electrode density is known to reduce the capacitance of the dielectric^71,72^. However, the increasing field localization with decreasing electrode density compensates this effect to provide overlapping curves for actuation in the low-strain region. In the high-strain region (> 0.1 mm/mm), the field localization increases from its low-field value, and the different electrode densities begin to exhibit different trends in the strain vs max E-field. In this region, higher values of the fiber density provide larger actuation strains.

Device failure occurs when any node in the simulation reaches the electrical breakdown field of the material. For devices with monotonic e_t_ vs $\widetilde{E}$, the failure of the device occurs when the ${\text{max}}(E)$ exceeds the electrical breakdown field of the material. However, if the device exhibits instability before reaching ${\text{max}}(E)$, the device will fail at the instability point. The material breakdown field of common dielectric elastomers falls in the range of 50 V/μm to 300 V/μm^73^ and is highly dependent on the thickness of the dielectric. For a PDMS film with a thickness of 0.3 mm, the theoretical breakdown field can be as high as ~ 145 V/μm^73^. Within the set of conditions in Fig. 2, the device with 2r/d = 0.333 fails due to instability at a strain of 36.3%, while the devices with smaller values of the fiber spacing fail due to electrical breakdown caused by field localization. Actuation metrics as a function of 2r/d for different values of h/d are compiled in Fig. S1.

### Effect of dielectric thickness (h/d)

**Figure 3:**
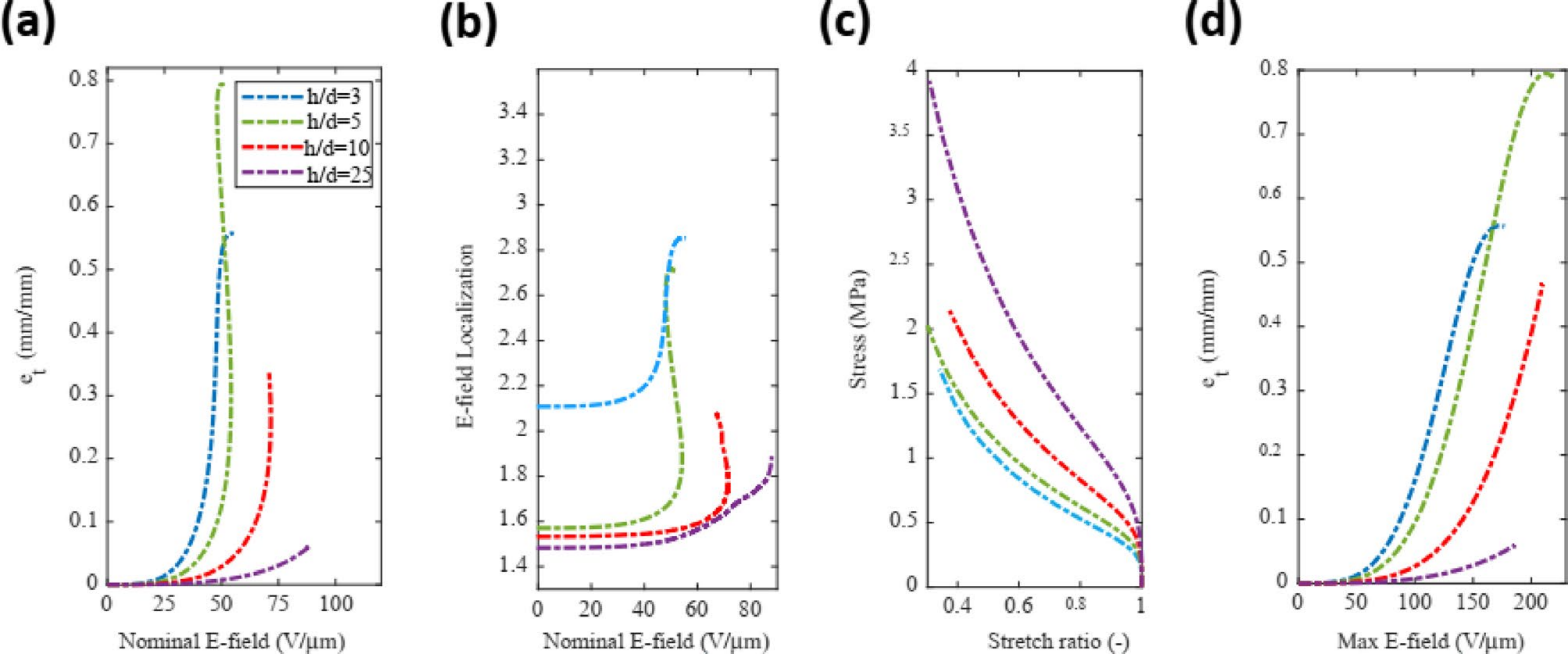
Actuation of a representative volume element with a fixed electrode density (2r/d = 0.33) and varying dielectric thickness. (**a**) Thickness actuation strain. (**b**) Field localization (E_max_/E_bulk_). (**c**) Compressive stress–strain behavior of the unit cells. (**d**) Strain as a function of the maximum E-field.

Figure 3 illustrates the effect of varying dielectric thickness (h/d) while keeping the fiber density constant at 2r/d = 0.333. As the dielectric thickness increases, the slope of the e_t_ vs $\widetilde{E}$ decreases (Fig. 3a). The E-field localization has little dependence on the dielectric thickness (Fig. 3b). However, as the dielectric thickness increases, the mechanical stiffness of the unit cell increases (Fig. 3c). Since the E-field localization is nearly independent of the dielectric thickness, the overall trend in the actuation vs ${\text{max}}(E)$ is dominated by the mechanics, resulting in a decrease in the actuation strain as the dielectric thickness increases (Fig. 3d).

### Parametric evaluation

**Figure 4:**
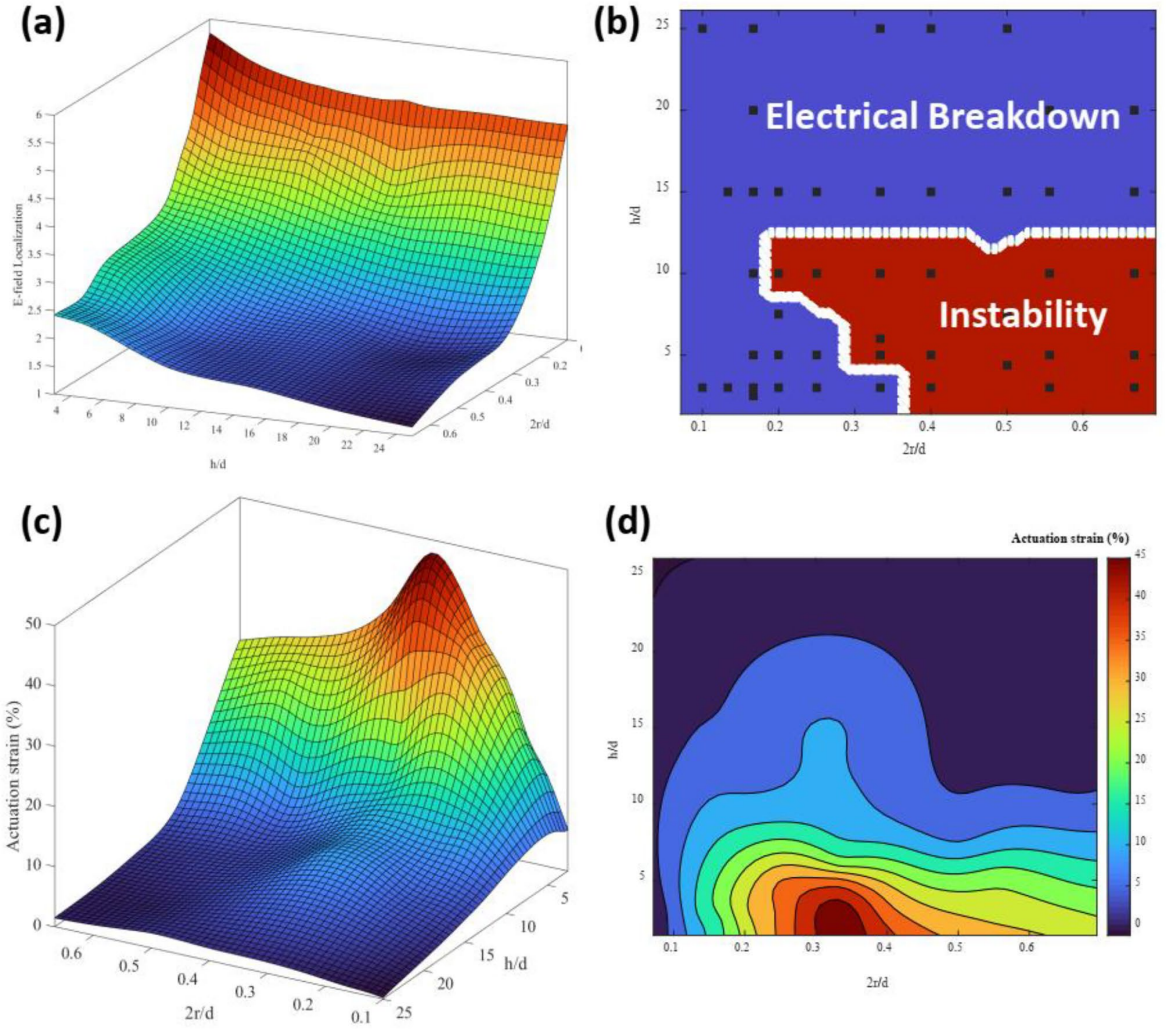
Parametric evaluation of device performance as a function of fiber density (2r/d) and dielectric thickness (h/d). (**a**) 3D surface plot representing the effect of fiber density (2r/d) and dielectric thickness (h/d) on the E-field localization, (**b**) failure mechanism of the actuators, regions shaded in blue represent failure due to the electrical breakdown (EB), regions shaded in red represent failure due to the electromechanical instability (EMI), (**c**) contour plot of the actuation strain dependency to the fiber density (2r/d) and fiber spacing (h/d) values, (**d**) 3D surface plot representing the effect of fiber density (2r/d) and dielectric thickness (h/d) on the DEA actuation strain.

Figure 4 compiles the results of a parametric study over a range of 2r/d from 0.667 to 0.1 and values of h/d ranging from 1.5 to 25. Similar to the trends observed in Figs. 2 and 3, the E-field localization depends strongly on the fiber density (2r/d) and depends weakly on the dielectric thickness (h/d), with monotonic trends in both parameters (Fig. 4a). The phase diagram indicating which devices fail due to instability and which devices fail due to electrical breakdown is shown in Fig. 4b when the breakdown field is assumed to be 145 V/μm. Electromechanical instability occurs in the range of high fiber density and low dielectric thickness. This correlates with the explanation that the instability is caused by changes in the E-field localization during actuation; the slope of the E-field vs geometric parameters (2r/d and h/d) is steepest in this region. The actuation strain at failure (Fig. 4c,d) has a peak near the value of 2r/d = 0.333 and h/d < 5. Instability occurs at higher values of 2r/d, while the actuation strain is limited by mechanics at higher values of h/d.

### Comparison to control device structures

**Figure 5:**
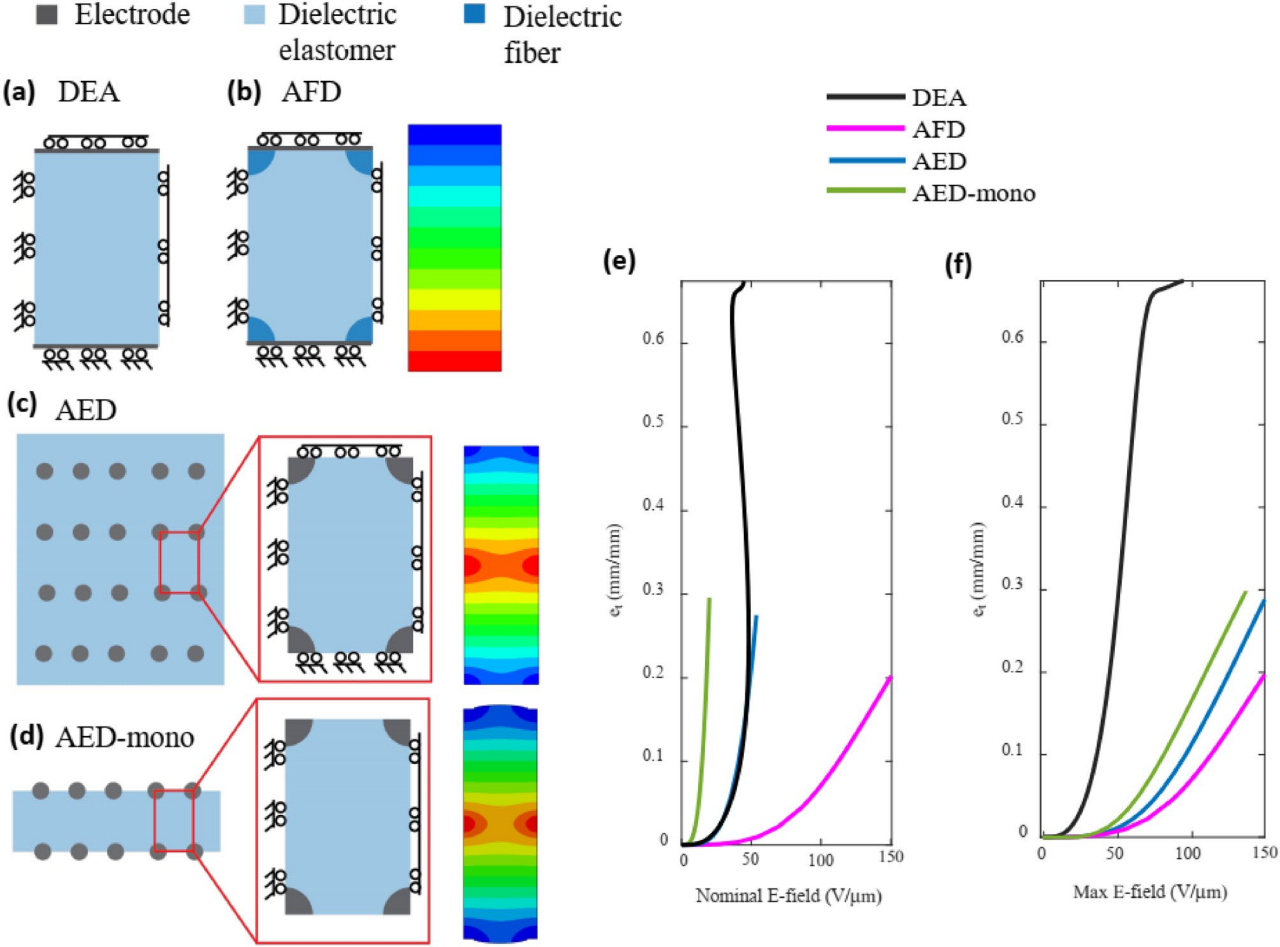
Comparison between different device structures. (**a**) Boundary conditions for a traditional soft DEA, (**b**) boundary conditions and voltage distribution for a DEA with stiff dielectric fibers (AFD), (**c**) boundary conditions and voltage distribution for a multilayer DEA with stiff electrode fibers (AED), (**d**) boundary conditions and voltage distribution for a monolayer DEA with stiff electrode fibers (AED). (**e**) Thickness strain as a function of the nominal electric field, (**f**) Thickness strain as a function of the max electric field in the dielectric.

Compared to traditional DEAs with continuous electrodes and isotropic mechanical properties, AED devices exhibit two key differences: (1) anisotropy in the mechanical properties, and (2) non-uniform electric field within the dielectric. To examine these effects independently, Fig. 5 compares several conditions. The control condition for a typical DEA with isotropic mechanical properties and uniform electric field (Fig. 5a) exhibits instability at 26% strain (Fig. 5e,f), which is consistent with the well-known analytical equation for Neo-Hookean materials (${\lambda }_{3}-{\lambda }_{3}^{4}=\frac{{\epsilon }_{0}{\epsilon }_{0}}{\mu }{\widetilde{E}}^{2}$)^74^. In the second control condition, fibers are treated as insulating dielectric materials with the same dielectric constant as the dielectric and the electrodes are assigned to be planar surfaces on the z-faces of the unit cell (Fig. 5b). This condition, referred to as aligned fiber DEA (AFD), provides anisotropy in the mechanical properties while retaining a uniform electric field. This AFD control was created using the same unit cell geometry as the optimal performance of AED (2r/d = 0.33 and h/d = 3). The AFD exhibits failure due to electrical breakdown at a strain of 19%.

A final control condition was implemented to compare the difference between a multilayer AED and a single layer AED. Periodic boundary conditions in all directions (all simulations shown above) approximate a multilayer AED. To investigate the behavior of a device with a single layer dielectric, the periodic boundary in the z direction was removed. In this situation, the concentration of the electric field between the electrodes causes the dielectric to locally actuate in the region between the electrodes, accelerating the actuation-induced E-field localization.

### Estimation of sheet resistance

One of the motivations for investigating the use of fiber-type electrodes is the potential for improved sheet resistance that could improve the actuation speed and reduce the heat generation during actuation. Zhao et al. developed an analytical dynamic model of a DEA that takes into account both the mechanical losses and the electrical losses^37^. For typical resistance values of carbon nanotube electrodes, this model predicted an increase in the operating frequency from 20 to 300 Hz by reducing the RC time constant from 5 to 0.4 ms. In addition to operation frequency, the electrode resistance impacts the efficiency and heat generation. Recent work on dielectric elastomer generators indicates that reducing sheet resistance using liquid metals can reduce the electrical power loss by 99%^75^. However, these liquid metals are difficult to incorporate into multilayer devices due to their liquidity. Lastly, resistive losses while operating DEAs at high frequencies can generate thermal energy that cause the device to overheat, which is a practical limitation on the flight time of flying robots powered by DEAs^12^.

The theoretical sheet resistance in aligned fiber electrodes is estimated by converting the cross-sectional area of the fiber electrodes to an equivalent electrode thickness. An approximation of these sheet resistances are shown in Supplementary Note 2. For a device with 2r/d = 0.333 and h/d = 3, the electrode radius (r) is h/18. The fiber area is $A=\pi {r}^{2}$ over a length of d. The equivalent thickness is then $t=\pi {r}^{2}/d$. The sheet resistance (R_s_) is related to the resistivity through ${R}_{s}=\rho /t$. The resistivity of silver is 15.6 × 10^–9^ Ω m. Assuming a metal-based composite with a resistivity 10 times higher than silver, the resistivity of the fibers could be 156 × 10^–9^ Ω·m. For a device with a dielectric thickness (h) of 40 μm, the optimal fiber distribution (2r/d = 0.333 and h/d = 3) gives a fiber radius of 2.22 μm. Assuming the electrode material has a conductivity ~ 10% of bulk silver, the effective sheet resistance would be ~ 0.136 Ω/□ (Fig. S3), which is several orders of magnitude lower than what can be achieved with carbon-based electrodes^76^.

## Discussion

We have used a dynamic finite element simulation approach to investigate the parameter space for dielectric elastomer actuators (DEAs) in which the electrodes are composed of aligned stiff fibers. Our parametric study identifies that the electric field localization is primarily determined by the fiber density within the electrodes while the device stiffness is determined by the dielectric thickness relative to the fiber density. The optimal fiber density and dielectric thickness is in the range of 2r/d ~ 0.33 and h/d ~ 3. Estimation of the sheet resistance based on common materials properties for metal composites indicate that these devices are expected to have RC time constants that are several orders of magnitude lower than conventional DEAs with low-stiffness electrodes composed of nanomaterials.

The control conditions in Fig. 5 show that, relative to a conventional homogeneous DEA, uniaxial mechanical constraints increase the voltage at which the device actuates to a given thickness strain. However, the electric field localization in the AED devices reduce the field at which the devices reach a given strain. Since the actuation strain in an electrostatic actuator depends on the square of the electric field, inhomogeneities in the field can lead to increased actuation even if the average field remains constant within a dielectric. In a typical device, inhomogeneous electric fields may cause local actuation and therefore accelerated electrical breakdown in the devices. The rational design of the structure and the periodic nature of the device prevent this local actuation and accelerated electrical breakdown. Consequently, this suggests that inhomogeneous electric fields may be beneficial in rationally-designed structures in which mechanical constraints prevent actuation localization despite the presence of electric field localization.

When preparing DEAs, the typical approach is to minimize the stiffness of the electrode materials. Our simulations of AED devices indicate that under the specific conditions investigated here, stiff electrodes can exhibit improved actuation performance compared to soft electrodes. However, our FEM simulations include some idealized assumptions. For example, our definition of the unit cell assumes continuous, no-slip mechanical boundaries between the dielectric and electrodes. Many materials such as carbon nanotubes and silver nanowires typically exhibit sliding at the interface between the electrode and the polymer^77^. Achieving the AED geometry in practical devices will require careful selection of the fabrication approach and interface engineering strategy to ensure sufficient interfacial strength between the electrodes and dielectric. Our current work analyzes the situation in which electrodes are oriented in the y-direction and aligned in the z-direction. This alignment in the z-direction between layers may be challenging to fabricate practically. Future work will investigate stacked layer geometries in which the electrode layers have offsets in the x-direction. Our AED stimulations approximate a device that is infinite in all directions due to the periodic boundary conditions. However, especially for multilayer structures, breakdown typically occurs at the edges of the electrodes^78^. The influence of fiber-type electrodes on these edge effects will require computational and experimental investigation. Lastly, as the electrode resistance decreases, the contact resistance between the fiber electrodes and the external electrical connections may become a challenge. Contact resistance can be minimized by adding electrode contacts during the dielectric multilayering process.

Electrostatic actuators occupy a unique niche in the field of soft actuators due to their fast actuation. Rationally designed electrodes that reduce the resistive losses and further increase the actuation frequency could enhance the key advantage of DEAs compared to other soft actuators and enable new application spaces. The fastest DEAs operate in the range of a few kHz^79,80^. Increasing the operation frequency to the range of tens of kHz could enable applications in wearable ultrasound^81^ medical devices and high-endurance microrobotics. While the actuation rate of electrohydraulic actuators is limited by the viscosity of the liquid^82^, these devices may benefit from other aspects of aligned electrodes, including the reduce operation voltage and the uniaxial stretchability.

## Methods

The actuation of DEAs can be modeled employing the balance of momentum and Gauss’s flux theorem (which can be derived directly from the Coulombs law)^60,83,84^. The two equations in the differential form are expressed over the domain as:

1 $$\begin{array}{*{20}l} {\frac{{\partial \sigma_{ij} }}{{\partial x_{i} }} + b_{j} = \rho a_{j} , j = 1,2,3} \hfill \\ {\frac{{\partial D_{i} }}{{\partial x_{i} }} = q } \hfill \\ \end{array} ,$$

where ${\sigma }_{ij}$ is Cauchy stress tensor components, ${b}_{j}$ and ${a}_{j}$ denote the body force and acceleration vector components, $\rho$ is the density, ${D}_{i}$ is electric displacement, and $q$ is the density of free charges. In this context to account for the contribution of the electrostatic forces, they may be considered as the body forces:

2 $${{\text{b}}}_{{\text{j}}}=\frac{\partial {\upsigma }_{{\text{ij}}}^{{\text{Maxwell}}}}{\partial {{\text{x}}}_{{\text{i}}}},$$

where ${\sigma }_{ij}^{Maxwell}$ stands for Maxwell stress tensor components. Based on the nonlinear theory of electroelasticity, it can be written as:

3 $${\sigma }_{ij}^{Maxwell}={\varepsilon }_{0}{\varepsilon }_{r}\left({E}_{i}{E}_{j}-\frac{1}{2}{E}_{k}{E}_{k}{\delta }_{ij}\right),$$

in which ${E}_{i}{, \varepsilon }_{0}$ and ${\varepsilon }_{r}$ are electric field components, vacuum permittivity and dielectric constant of the elastomer.

There is a linear relationship between ${D}_{i}$ and ${E}_{i}$, for vacuum or non-polarizable materials:

4 $${D}_{i}={\varepsilon }_{0}{\varepsilon }_{r}{E}_{i}.$$

Supplementary Note 1 provides the detailed mathematical steps in the development of the weak form of these equations that is used in the finite element formulation of the UEL in ABAQUS software.

Dynamic simulations with a voltage ramp were performed until the simulation failed. Information extracted from the simulations includes the actuation strain and the degree of uniformity of the electric field within the dielectric. A parameterized simulation is set up to examine these factors as a function of dimensionless parameters (d/r and h/d). The structure has been meshed using an 8-node thermally coupled brick, trilinear displacement and temperature, reduced integration, and hourglass control (C3D8RT). Mesh and increment studies also were conducted to ensure the convergence of the present numerical model. The total number of elements used in the model is 148,552.

### Supplementary Information

Supplementary Information.

## Data Availability

The data that support the findings of this study are available from the corresponding author upon reasonable request.
