# Supplementary material for: In silico optimization of aligned fiber electrodes for dielectric elastomer actuators
Source: Sci Rep. 2024 Feb 27;14:4703. doi: 10.1038/s41598-024-54931-y (PMC10897417; doi:10.1038/s41598-024-54931-y)
Supplement: Supplementary file 1 — Supplementary Information. [file 41598_2024_54931_MOESM1_ESM.pdf]

Fiber-Type Electrodes for Improved Actuation of Dielectric Elastomer Actuators

Supplementary Information

Supplementary Note 1: Dynamic finite element analysis of dielectric elastomer actuators

Actuation of dielectric elastomers can be modelled using the balance of momentum and the Gauss's flux theorem (which can be derived directly from the Coulombs law). The two equations in differential form are expressed over the domain as

$$\begin{gathered}\frac{\partial \sigma_{ij}}{\partial x_i} + b_j = \rho a_j, \qquad j = 1,2,3 \\ \frac{\partial D_i}{\partial x_i} = q\end{gathered} \tag{A1}$$

where σ_{ij} is Cauchy stress tensor components, b_j and a_j denote the body force and acceleration vector components, ρ is the density, D_i is electric displacement, and q is the density of free charges. In this context to account for the contribution of the electrostatic forces, they may be considered as the body forces:

$$b_j = \frac{\partial \sigma_{ij}^{Maxwell}}{\partial x_i} \tag{A2}$$

where $\sigma_{ij}^{Maxwell}$ stands for Maxwell stress tensor components. Based on the nonlinear theory of electroelasticity, it can be written as:

$$\sigma_{ij}^{Maxwell} = \varepsilon_0 \varepsilon_r (E_i E_j - \frac{1}{2} E_k E_k \delta_{ij}) \tag{A3}$$

There is a linear relationship between D_i and E_i, for vacuum or non-polarizable materials:

$$D_i = \varepsilon_0 \varepsilon_r E_i \tag{A4}$$

It can be shown that these differential equations (eq. 1) are equivalent to the following integral forms.

$$\begin{gathered}\int_V \left(\frac{\partial \sigma_{ij}}{\partial x_i} + b_j - \rho a_j \right) w_{1j} dV = 0, \qquad \forall w_{1j}, \qquad j = 1,2,3 \\ \int_V \left(\frac{\partial D_i}{\partial x_i} - q \right) w_2 dV = 0, \qquad \forall w_2\end{gathered} \tag{A5}$$

where w_{1j} and w_2 are weight functions and can be any arbitrary test functions.

Using integration by parts we can modify these two equations such that they better suit in the context of finite element analysis. For the balance of momentum we have:

$$\begin{aligned}&\frac{\partial \sigma_{ij}}{\partial x_i} w_{1j} = \frac{\partial}{\partial x_i}(\sigma_{ij} w_{1j}) - \sigma_{ij} \frac{\partial w_{1j}}{\partial x_i} \\ &\to \int_V \left(\frac{\partial \sigma_{ij}}{\partial x_i} + b_j - \rho a_j \right) w_{1j} dV = \int_V \left(-\sigma_{ij} \frac{\partial w_{1j}}{\partial x_i} + b_j w_{1j} - \rho a_j w_{1j} \right) dV + \int_V \frac{\partial}{\partial x_i}(\sigma_{ij} w_{1j}) dV \\ &= \int_V \left(-\sigma_{ij} \frac{\partial w_{1j}}{\partial x_i} + b_j w_{1j} - \rho a_j w_{1j} \right) dV + \int_S w_{1j} \underbrace{\sigma_{ij} n_i}_{t_j} dS \\ &\to \int_V \left(-\sigma_{ij} \frac{\partial w_{1j}}{\partial x_i} + b_j w_{1j} - \rho a_j w_{1j} \right) dV + \int_S w_{1j} t_j dS = 0, \qquad j = 1,2,3\end{aligned} \tag{A6}$$

And for the Gauss's equation we have

$$\frac{\partial D_i}{\partial x_i} w_2 = \frac{\partial}{\partial x_i}(D_i w_2) - D_i \frac{\partial w_2}{\partial x_i}$$

$$\rightarrow \int_V \left(\frac{\partial D_i}{\partial x_i} - q\right) w_2 dV = \int_V \left(-D_i \frac{\partial w_2}{\partial x_i} - q w_2\right) dV + \int_V \frac{\partial}{\partial x_i}(D_i w_2) dV$$

$$= \int_V \left(-D_i \frac{\partial w_2}{\partial x_i} - q w_2\right) dV + \int_S w_2 \underbrace{D_i n_i}_{q_s} dS \qquad (A7)$$

$$\rightarrow \int_V \left(-D_i \frac{\partial w_2}{\partial x_i} - q w_2\right) dV + \int_S w_2 q_s dS = 0$$

Therefore, modelling of actuation of dielectric elastomers means solving for functions u_k and ϕ in the following system of four partial differential equations together with the given boundary and initial conditions.

$$\begin{Bmatrix} R_{u_j} \\ R_\phi \end{Bmatrix} = \begin{Bmatrix} \int_V \left(-\sigma_{ij} \frac{\partial w_{1j}}{\partial x_i} + b_j w_{1j} - \rho a_j w_{1j}\right) dV + \int_S w_{1j} t_j dS \\ \int_V \left(-D_i \frac{\partial w_2}{\partial x_i} - q w_2\right) dV + \int_S w_2 q_s dS \end{Bmatrix} = \begin{Bmatrix} 0 \\ 0 \end{Bmatrix}, \qquad j = 1,2,3 \qquad (A8)$$

That u_k , ϕ , t_j , R_{u_j} , R_ϕ are representing displacement, electric potential, surface traction vector, residual values of displacement and residual values of electric potential.

In finite elements approximation we relax the requirement that w_{1j} and w_2 can be any arbitrary functions and instead we consider a function shape for them, i.e.

$$w_{1j}(\xi_1, \xi_2, \xi_3) = N^A(\xi_1, \xi_2, \xi_3) w_{1j}^A, \qquad \forall w_{1j}^A, \qquad j = 1,2,3$$

$$w_2(\xi_1, \xi_2, \xi_3) = N^A(\xi_1, \xi_2, \xi_3) w_2^A, \qquad \forall w_2^A \qquad (A9)$$

where $N^A(\xi_1, \xi_2, \xi_3)$ are shape functions expressed in a local coordinate system ξ_1, ξ_2, ξ_3, and subscript A is the node number. Therefore:

$$\begin{Bmatrix} R_{u_j} \\ R_\phi \end{Bmatrix} = \begin{Bmatrix} \int_V \left(-\sigma_{ij} \frac{\partial N^A}{\partial x_i} w_{1j}^A + b_j N^A w_{1j}^A - \rho a_j N^A w_{1j}^A\right) dV + \int_S t_j N^A w_{1j}^A dS \\ \int_V \left(-D_i \frac{\partial N^A}{\partial x_i} w_2^A - q N^A w_2^A\right) dV + \int_S q_s N^A w_2^A dS \end{Bmatrix} = \begin{Bmatrix} 0 \\ 0 \end{Bmatrix}, \qquad (A10)$$

$$j = 1,2,3$$

Since this equation holds for any arbitrary w_{1j}^A and w_2^A, we can conclude that each term in the summation is zero, which results in the following system of $4N$ equations where N is the number of the nodes.

$$\begin{Bmatrix} R_{u_j}^A \\ R_\phi^A \end{Bmatrix} = \begin{Bmatrix} \int_V \left(-\sigma_{ij} \frac{\partial N^A}{\partial x_i} + b_j N^A - \rho a_j N^A\right) dV + \int_S t_j N^A dS \\ \int_V \left(-D_i \frac{\partial N^A}{\partial x_i} - q N^A\right) dV + \int_S q_s N^A dS \end{Bmatrix} = \begin{Bmatrix} 0 \\ 0 \end{Bmatrix}, \qquad j = 1,2,3, \qquad (A11)$$

$$A = 1, \ldots, N$$

In finite elements approximation we also need to use a shape function for the functions u_k and ϕ. This will turn this system of $4N$ partial differential equations with four unknown functions into a system of $4N$ nonlinear algebraic equations with $4N$ unknown variables.

$$u_k(\xi_1, \xi_2, \xi_3, t) = N^B(\xi_1, \xi_2, \xi_3) u_k^B(t) \qquad (A12)$$

$$\phi(\xi_1,\xi_2,\xi_3,t) = N^B(\xi_1,\xi_2,\xi_3)\phi^B(t)$$

For geometrical discretization we also use the same shape function as the displacement, i.e.

$$\begin{aligned} X_k(\xi_1,\xi_2,\xi_3) &= N^B(\xi_1,\xi_2,\xi_3)X_k^B \\ x_k(\xi_1,\xi_2,\xi_3,t) &= X_k(\xi_1,\xi_2,\xi_3) + u_k(\xi_1,\xi_2,\xi_3) = N^B(\xi_1,\xi_2,\xi_3)\left(X_k^B + u_k^B(t)\right) \\ &= N^B(\xi_1,\xi_2,\xi_3)x_k^B(t) \end{aligned} \quad (A13)$$

To evaluate the integrals we may use the Gaussian quadrature which results in

$$\begin{Bmatrix} R_{u_j}^A \\ R_\phi^A \end{Bmatrix} = \begin{Bmatrix} \sum_{n_G}\left(-\sigma_{ij}\frac{\partial N^A}{\partial x_i} + b_j N^A - \rho a_j N^A\right) w_{n_G} \det\frac{\partial x_p}{\partial \xi_q} + \sum_{n_G}\left(t_j N^A\right) w_{n_G} \det\frac{\partial x_p}{\partial \xi_q} \\ \sum_{n_G}\left(-D_i\frac{\partial N^A}{\partial x_i} - qN^A\right) w_{n_G} \det\frac{\partial x_p}{\partial \xi_q} + \sum_{n_G}(q_s N^A) w_{n_G} \det\frac{\partial x_p}{\partial \xi_q} \end{Bmatrix} \quad (A14)$$

This set of nonlinear algebraic equations can be solved iteratively using Newton-Raphson's method as

$$\begin{aligned} &\begin{Bmatrix} R_{u_j}^A \\ R_\phi^A \end{Bmatrix}^{i+1} = \begin{Bmatrix} R_{u_j}^A \\ R_\phi^A \end{Bmatrix}^{i} + \frac{\partial}{\partial u_k^B}\begin{Bmatrix} R_{u_j}^A \\ R_\phi^A \end{Bmatrix}^{i} \delta u_k^B + \frac{\partial}{\partial \phi^B}\begin{Bmatrix} R_{u_j}^A \\ R_\phi^A \end{Bmatrix}^{i} \delta\phi^B = \begin{Bmatrix} 0 \\ 0 \end{Bmatrix}, \qquad j = 1,2,3, \\ &\qquad A = 1,\dots,N \\ &\rightarrow -\frac{\partial}{\partial u_k^B}\begin{Bmatrix} R_{u_j}^A \\ R_\phi^A \end{Bmatrix}\delta u_k^B - \frac{\partial}{\partial \phi^B}\begin{Bmatrix} R_{u_j}^A \\ R_\phi^A \end{Bmatrix}\delta\phi^B = \begin{Bmatrix} R_{u_j}^A \\ R_\phi^A \end{Bmatrix}, \qquad j = 1,2,3, \qquad A = 1,\dots,N \end{aligned} \quad (A15)$$

where the superscripts $i+1$ and i are the iteration number. Therefore

$$\begin{bmatrix} K_{u_j^A u_k^B} & K_{u_j^A \phi^B} \\ K_{\phi^A u_k^B} & K_{\phi^A \phi^B} \end{bmatrix} \begin{Bmatrix} \delta u_k^B \\ \delta\phi^B \end{Bmatrix} = \begin{Bmatrix} R_{u_j}^A \\ R_\phi^A \end{Bmatrix}, \qquad j = 1,2,3, \qquad A = 1,\dots,N \quad (A16)$$

where

$$\begin{aligned} K_{u_j^A u_k^B} &= -\frac{\partial}{\partial u_k^B}\left(\sum_{n_G}\left(-\sigma_{ij}\frac{\partial N^A}{\partial x_i} + b_j N^A - \rho a_j N^A\right) w_{n_G} \det\frac{\partial x_p}{\partial \xi_q} + \sum_{n_G}\left(t_j N^A\right) w_{n_G} \det\frac{\partial x_p}{\partial \xi_q}\right) \\ K_{u_j^A \phi^B} &= -\frac{\partial}{\partial \phi^B}\left(\sum_{n_G}\left(-\sigma_{ij}\frac{\partial N^A}{\partial x_i} + b_j N^A - \rho a_j N^A\right) w_{n_G} \det\frac{\partial x_p}{\partial \xi_q} + \sum_{n_G}\left(t_j N^A\right) w_{n_G} \det\frac{\partial x_p}{\partial \xi_q}\right) \\ K_{\phi^A u_k^B} &= -\frac{\partial}{\partial u_k^B}\left(\sum_{n_G}\left(-D_i\frac{\partial N^A}{\partial x_i} - qN^A\right) w_{n_G} \det\frac{\partial x_p}{\partial \xi_q} + \sum_{n_G}(q_s N^A) w_{n_G} \det\frac{\partial x_p}{\partial \xi_q}\right) \\ K_{\phi^A \phi^B} &= -\frac{\partial}{\partial \phi^B}\left(\sum_{n_G}\left(-D_i\frac{\partial N^A}{\partial x_i} - qN^A\right) w_{n_G} \det\frac{\partial x_p}{\partial \xi_q} + \sum_{n_G}(q_s N^A) w_{n_G} \det\frac{\partial x_p}{\partial \xi_q}\right) \end{aligned} \quad (A17)$$

Stress is often expressed in terms of the gradient deformation tensor, and therefore it's easier to take the derivative with respect to the gradient deformation tensor rather than the nodal variables u_k^B and ϕ^B.

$$
\begin{aligned}
F_{nm} &= \frac{\partial x_n}{\partial X_m} = \frac{\partial}{\partial X_m}(N^A x_n^A) = \frac{\partial N^A}{\partial X_m} x_n^A \\
\rightarrow \frac{\partial}{\partial u_k^B}(\square) &= \frac{\partial F_{nm}}{\partial u_k^B}\frac{\partial}{\partial F_{nm}}(\square) = \frac{\partial N^A}{\partial X_m}\delta_{nk}\delta_{AB}\frac{\partial}{\partial F_{nm}}(\square) = \frac{\partial N^B}{\partial X_m}\frac{\partial}{\partial F_{km}}(\square) \\
&= \frac{\partial N^B}{\partial x_l}\frac{\partial x_l}{\partial X_m}\frac{\partial}{\partial F_{km}}(\square) = \frac{\partial N^B}{\partial x_l}F_{lm}\frac{\partial}{\partial F_{km}}(\square)
\end{aligned}
\qquad (A18)
$$

Now we only need to evaluate the components of the stiffness matrix. For $K_{u_j^A u_k^B}$ we have

$$
\begin{aligned}
K_{u_j^A u_k^B} &= -\frac{\partial}{\partial u_k^B}\left(\left(-\sigma_{ij}\frac{\partial N^A}{\partial x_i} + b_j N^A - \rho a_j N^A\right) w_{n_G} \det\frac{\partial x_p}{\partial \xi_q}\right) \\
&= -\frac{\partial N^B}{\partial x_l}F_{lm}\frac{\partial}{\partial F_{km}}\left(\left(-\sigma_{ij}\frac{\partial N^A}{\partial x_i} + b_j N^A - \rho a_j N^A\right) w_{n_G} \det\frac{\partial x_p}{\partial \xi_q}\right) \\
&= \frac{\partial N^B}{\partial x_l}F_{lm}\frac{\partial}{\partial F_{km}}\left(\sigma_{ij}\frac{\partial N^A}{\partial x_i} w_{n_G} \det\frac{\partial x_p}{\partial \xi_q}\right) - \frac{\partial N^B}{\partial x_l}F_{lm}\frac{\partial}{\partial F_{km}}\left(b_j N^A w_{n_G} \det\frac{\partial x_p}{\partial \xi_q}\right) \\
&+ \frac{\partial N^B}{\partial x_l}F_{lm}\frac{\partial}{\partial F_{km}}\left(\rho a_j N^A w_{n_G} \det\frac{\partial x_p}{\partial \xi_q}\right)
\end{aligned}
\qquad (A19)
$$

For the first term in this equation we have

$$
\begin{aligned}
&\frac{\partial N^B}{\partial x_l}F_{lm}\frac{\partial}{\partial F_{km}}\left(\sigma_{ij}\frac{\partial N^A}{\partial x_i} w_{n_G} \det\frac{\partial x_p}{\partial \xi_q}\right) \\
&\quad = \frac{\partial N^B}{\partial x_l}F_{lm}\frac{\partial \sigma_{ij}}{\partial F_{km}}\frac{\partial N^A}{\partial x_i} w_{n_G} \det\frac{\partial x_p}{\partial \xi_q} + \frac{\partial N^B}{\partial x_l}F_{lm}\sigma_{ij}\frac{\partial}{\partial F_{km}}\left(\frac{\partial N^A}{\partial x_i}\right) w_{n_G} \det\frac{\partial x_p}{\partial \xi_q} \\
&\quad + \frac{\partial N^B}{\partial x_l}F_{lm}\sigma_{ij}\frac{\partial N^A}{\partial x_i} w_{n_G}\frac{\partial}{\partial F_{km}}\left(\det\frac{\partial x_p}{\partial \xi_q}\right)
\end{aligned}
\qquad (A20)
$$

Knowing that $\frac{\partial F_{ni}^{-1}}{\partial F_{km}} = -F_{nk}^{-1}F_{mi}^{-1}$, for the second term of this equation we have

$$
\begin{aligned}
\frac{\partial N^B}{\partial x_l}F_{lm}\sigma_{ij}\frac{\partial}{\partial F_{km}}\left(\frac{\partial N^A}{\partial x_i}\right) w_{n_G} \det\frac{\partial x_p}{\partial \xi_q} &= \frac{\partial N^B}{\partial x_l}F_{lm}\sigma_{ij}\frac{\partial}{\partial F_{km}}\left(\frac{\partial N^A}{\partial X_n}\frac{\partial X_n}{\partial x_i}\right) w_{n_G} \det\frac{\partial x_p}{\partial \xi_q} \\
&= \frac{\partial N^B}{\partial x_l}F_{lm}\sigma_{ij}\frac{\partial}{\partial F_{km}}\left(\frac{\partial N^A}{\partial X_n}F_{ni}^{-1}\right) w_{n_G} \det\frac{\partial x_p}{\partial \xi_q} \\
&= -\frac{\partial N^B}{\partial x_l}F_{lm}\sigma_{ij}\frac{\partial N^A}{\partial X_n}F_{nk}^{-1}F_{mi}^{-1} w_{n_G} \det\frac{\partial x_p}{\partial \xi_q} \\
&= -\frac{\partial N^B}{\partial x_l}\sigma_{ij}\frac{\partial N^A}{\partial X_n}\frac{\partial X_n}{\partial x_k}\delta_{li} w_{n_G} \det\frac{\partial x_p}{\partial \xi_q} = -\frac{\partial N^B}{\partial x_l}\sigma_{lj}\frac{\partial N^A}{\partial x_k} w_{n_G} \det\frac{\partial x_p}{\partial \xi_q} \\
&= -\frac{\partial N^B}{\partial x_l}\delta_{ik}\sigma_{lj}\frac{\partial N^A}{\partial x_i} w_{n_G} \det\frac{\partial x_p}{\partial \xi_q}
\end{aligned}
\qquad (A21)
$$

For the third term one may write

$$\frac{\partial N^B}{\partial x_l}F_{lm}\sigma_{ij}\frac{\partial N^A}{\partial x_i}w_{n_G}\frac{\partial}{\partial F_{km}}\left(\det\frac{\partial x_p}{\partial \xi_q}\right)=\frac{\partial N^B}{\partial x_l}F_{lm}\sigma_{ij}\frac{\partial N^A}{\partial x_i}w_{n_G}\frac{\partial}{\partial F_{km}}\left(\det\frac{\partial x_p}{\partial X_n}\det\frac{\partial X_n}{\partial \xi_q}\right)=$$

$$=\frac{\partial N^B}{\partial x_l}F_{lm}\sigma_{ij}\frac{\partial N^A}{\partial x_i}w_{n_G}\frac{\partial}{\partial F_{km}}\left(\det F_{pn}\right)\det\frac{\partial X_n}{\partial \xi_q} \quad (A22)$$

$$=\frac{\partial N^B}{\partial x_l}F_{lm}\sigma_{ij}\frac{\partial N^A}{\partial x_i}w_{n_G}F^{-1}_{mk}\det F_{pn}\det\frac{\partial X_n}{\partial \xi_q}=\frac{\partial N^B}{\partial x_l}\delta_{lk}\sigma_{ij}\frac{\partial N^A}{\partial x_i}w_{n_G}\det\frac{\partial x_p}{\partial \xi_q}$$

Therefore, the first term of $K_{u_j^A u_k^B}$ can be simplified as

$$\frac{\partial N^B}{\partial x_l}F_{lm}\frac{\partial}{\partial F_{km}}\left(-\sigma_{ij}\frac{\partial N^A}{\partial x_i}w_{n_G}\det\frac{\partial x_p}{\partial \xi_q}\right)$$

$$=\frac{\partial N^B}{\partial x_l}\left(F_{lm}\frac{\partial \sigma_{ij}}{\partial F_{km}}+\delta_{lk}\sigma_{ij}-\delta_{ik}\sigma_{lj}\right)\frac{\partial N^A}{\partial x_i}w_{n_G}\det\frac{\partial x_p}{\partial \xi_q} \quad (A23)$$

$$A=1,\dots,N$$

For the first term we have

$$\sigma_{ij}^{Max}=\epsilon\left(E_iE_j-\frac{1}{2}E_nE_n\delta_{ij}\right)=\epsilon\left(\frac{\partial\phi}{\partial x_i}\frac{\partial\phi}{\partial x_j}-\frac{1}{2}\frac{\partial\phi}{\partial x_n}\frac{\partial\phi}{\partial x_n}\delta_{ij}\right)$$

$$=\epsilon\left(\frac{\partial\phi}{\partial X_q}\frac{\partial X_q}{\partial x_i}\frac{\partial\phi}{\partial X_r}\frac{\partial X_r}{\partial x_j}-\frac{1}{2}\frac{\partial\phi}{\partial X_q}\frac{\partial X_q}{\partial x_n}\frac{\partial\phi}{\partial X_r}\frac{\partial X_r}{\partial x_n}\delta_{ij}\right) \quad (A24.1)$$

$$=\epsilon\left(\frac{\partial\phi}{\partial X_q}F^{-1}_{qi}\frac{\partial\phi}{\partial X_r}F^{-1}_{rj}-\frac{1}{2}\frac{\partial\phi}{\partial X_q}F^{-1}_{qn}\frac{\partial\phi}{\partial X_r}F^{-1}_{rn}\delta_{ij}\right)$$

$$\rightarrow\frac{\partial\sigma_{ij}^{Max}}{\partial F_{km}}=-\epsilon\left(\frac{\partial\phi}{\partial X_q}F^{-1}_{qk}F^{-1}_{mi}\frac{\partial\phi}{\partial X_r}F^{-1}_{rj}+\frac{\partial\phi}{\partial X_q}F^{-1}_{qi}\frac{\partial\phi}{\partial X_r}F^{-1}_{rk}F^{-1}_{mj}-\frac{1}{2}\frac{\partial\phi}{\partial X_q}F^{-1}_{qk}F^{-1}_{mn}\frac{\partial\phi}{\partial X_r}F^{-1}_{rn}\delta_{ij}\right.$$
$$\left.-\frac{1}{2}\frac{\partial\phi}{\partial X_q}F^{-1}_{qn}\frac{\partial\phi}{\partial X_r}F^{-1}_{rk}F^{-1}_{mn}\delta_{ij}\right) \quad (A24.2)$$

$$\rightarrow F_{lm}\frac{\partial\sigma_{ij}^{Max}}{\partial F_{km}} = -\epsilon\left(\frac{\partial\phi}{\partial X_q}F_{qk}^{-1}\delta_{li}\frac{\partial\phi}{\partial X_r}F_{rj}^{-1} + \frac{\partial\phi}{\partial X_q}F_{qi}^{-1}\frac{\partial\phi}{\partial X_r}F_{rk}^{-1}\delta_{lj} - \frac{1}{2}\frac{\partial\phi}{\partial X_q}F_{ql}^{-1}\frac{\partial\phi}{\partial X_r}F_{rk}^{-1}\delta_{ln}\delta_{ij} - \frac{1}{2}\frac{\partial\phi}{\partial X_q}F_{qk}^{-1}\delta_{ln}\frac{\partial\phi}{\partial X_r}F_{rl}^{-1}\delta_{ij}\right)$$
$$= -\epsilon\left(\frac{\partial\phi}{\partial X_q}\frac{\partial X_q}{\partial x_k}\delta_{li}\frac{\partial\phi}{\partial X_r}\frac{\partial X_r}{\partial x_j} + \frac{\partial\phi}{\partial X_q}\frac{\partial X_q}{\partial x_i}\frac{\partial\phi}{\partial X_r}\frac{\partial X_r}{\partial x_k}\delta_{lj} - \frac{1}{2}\frac{\partial\phi}{\partial X_q}\frac{\partial X_q}{\partial x_l}\frac{\partial\phi}{\partial X_r}\frac{\partial X_r}{\partial x_k}\delta_{ln}\delta_{ij} - \frac{1}{2}\frac{\partial\phi}{\partial X_q}\frac{\partial X_q}{\partial x_k}\delta_{ln}\frac{\partial\phi}{\partial X_r}\frac{\partial X_r}{\partial x_l}\delta_{ij}\right) \quad (A24.3)$$
$$= -\epsilon\left(\frac{\partial\phi}{\partial x_k}\delta_{li}\frac{\partial\phi}{\partial x_j} + \frac{\partial\phi}{\partial x_i}\frac{\partial\phi}{\partial x_k}\delta_{lj} - \frac{1}{2}\frac{\partial\phi}{\partial x_l}\frac{\partial\phi}{\partial x_k}\delta_{ln}\delta_{ij} - \frac{1}{2}\frac{\partial\phi}{\partial x_k}\delta_{ln}\frac{\partial\phi}{\partial x_l}\delta_{ij}\right)$$
$$= \epsilon\left(-E_k\delta_{li}E_j - E_iE_k\delta_{lj} + \frac{1}{2}E_lE_k\delta_{ln}\delta_{ij} + \frac{1}{2}E_k\delta_{ln}E_l\delta_{ij}\right)$$
$$= \epsilon\left(-E_kE_j\delta_{li} - E_iE_k\delta_{lj} + E_lE_k\delta_{ij}\right)$$

$$\rightarrow \frac{\partial N^B}{\partial x_l}F_{lm}\frac{\partial\sigma_{ij}^{Max}}{\partial F_{km}}\frac{\partial N^A}{\partial x_i}w_{n_G}\det\frac{\partial x_p}{\partial\xi_q} = \frac{\partial N^B}{\partial x_l}\epsilon\left(-E_kE_j\delta_{li} - E_iE_k\delta_{lj} + E_lE_k\delta_{ij}\right)\frac{\partial N^A}{\partial x_i}w_{n_G}\det\frac{\partial x_p}{\partial\xi_q}$$
$$\rightarrow \frac{\partial N^B}{\partial x_l}F_{lm}\frac{\partial}{\partial F_{km}}\left(-\sigma_{ij}\frac{\partial N^A}{\partial x_i}w_{n_G}\det\frac{\partial x_p}{\partial\xi_q}\right) \quad (A24.4)$$
$$= \frac{\partial N^B}{\partial x_l}\epsilon\left(-E_kE_j\delta_{li} - E_iE_k\delta_{lj} + E_lE_k\delta_{ij} + E_iE_j\delta_{lk} - \frac{1}{2}E_mE_m\delta_{lk}\delta_{ij} - E_lE_j\delta_{ik} + \frac{1}{2}E_mE_m\delta_{ik}\delta_{lj}\right)\frac{\partial N^A}{\partial x_i}w_{n_G}\det\frac{\partial x_p}{\partial\xi_q}$$

The surface force term of $K_{u_j^Au_k^B}$ is

$$-\frac{\partial N^B}{\partial x_l}F_{lm}\frac{\partial}{\partial F_{km}}\left(b_jN^Aw_{n_G}\det\frac{\partial x_p}{\partial\xi_q}\right)$$
$$= -\frac{\partial N^B}{\partial x_l}F_{lm}\frac{\partial b_j}{\partial F_{km}}N^Aw_{n_G}\det\frac{\partial x_p}{\partial\xi_q} - \frac{\partial N^B}{\partial x_l}F_{lm}b_jN^Aw_{n_G}\frac{\partial}{\partial F_{km}}\left(\det\frac{\partial x_p}{\partial\xi_q}\right) \quad (A25)$$
$$= -\frac{\partial N^B}{\partial x_l}\left(F_{lm}\frac{\partial b_j}{\partial F_{km}} + \delta_{lk}b_j\right)N^Aw_{n_G}\det\frac{\partial x_p}{\partial\xi_q}$$

The inertia term of $K_{u_j^Au_k^B}$ is …

$$\rho = \frac{\rho_0}{\det\frac{\partial x_p}{\partial X_r}} = \rho_0\det\frac{\partial X_r}{\partial x_p} \rightarrow \frac{\partial}{\partial u_k^B}\left(\rho a_jN^Aw_{n_G}\det\frac{\partial x_p}{\partial\xi_q}\right) = \frac{\partial}{\partial u_k^B}\left(\rho_0a_jN^Aw_{n_G}\det\left(\frac{\partial X_r}{\partial x_p}\frac{\partial x_p}{\partial\xi_q}\right)\right)$$
$$= \rho_0\frac{\partial a_j}{\partial u_k^B}N^Aw_{n_G}\det\left(\frac{\partial X_r}{\partial\xi_q}\right) = \rho\frac{\partial a_j}{\partial u_k^B}N^Aw_{n_G}\det\frac{\partial x_p}{\partial\xi_q} \quad (A26)$$

Acceleration can be expressed as

$$a_j = \frac{1}{\beta\Delta t^2}\left(u_j - u_{0j} - v_{0j}\Delta t - \left(\frac{1}{2} - \beta\right)a_{0j}\Delta t^2\right) \rightarrow \frac{\partial a_j}{\partial u_k^B} = \frac{1}{\beta\Delta t^2}\frac{\partial u_j}{\partial u_k^B} = \frac{1}{\beta\Delta t^2}N^B \quad (A27)$$
$$v_j = v_{0j} + (1-\gamma)a_{0j}\Delta t + \gamma a_j\Delta t$$

$$\rightarrow \frac{\partial a_j}{\partial u_k^B} = \frac{\partial}{\partial u_k^B}\left(\frac{1}{\beta\Delta t^2}\left(N^C u_j^C - u_{0j} - v_{0j}\Delta t - \left(\frac{1}{2} - \beta\right) a_{0j}\Delta t^2\right)\right) = \frac{1}{\beta\Delta t^2} N^C \delta_{jk}\delta_{BC}$$

$$= \frac{1}{\beta\Delta t^2} N^B \delta_{jk}$$

$$\rightarrow \rho \frac{\partial a_j}{\partial u_k^B} N^A w_{n_G} \det \frac{\partial x_p}{\partial \xi_q} = \frac{1}{\beta\Delta t^2} \rho N^A N^B \delta_{jk} w_{n_G} \det \frac{\partial x_p}{\partial \xi_q}$$

Therefore, the stiffness terms that need to be evaluated inside the UEL are

$$K_{u_j^A u_k^B} = \left(\frac{\partial N^B}{\partial x_l} \epsilon \left(-E_k E_j \delta_{li} - E_i E_k \delta_{lj} + E_l E_k \delta_{ij} + E_i E_j \delta_{lk} - \frac{1}{2} E_m E_m \delta_{lk}\delta_{ij} - E_l E_j \delta_{ik} + \frac{1}{2} E_m E_m \delta_{ik}\delta_{lj}\right) \frac{\partial N^A}{\partial x_i} + \frac{1}{\beta\Delta t^2} \rho N^A N^B \delta_{jk}\right) w_{n_G} \det \frac{\partial x_p}{\partial \xi_q} \qquad (A28)$$

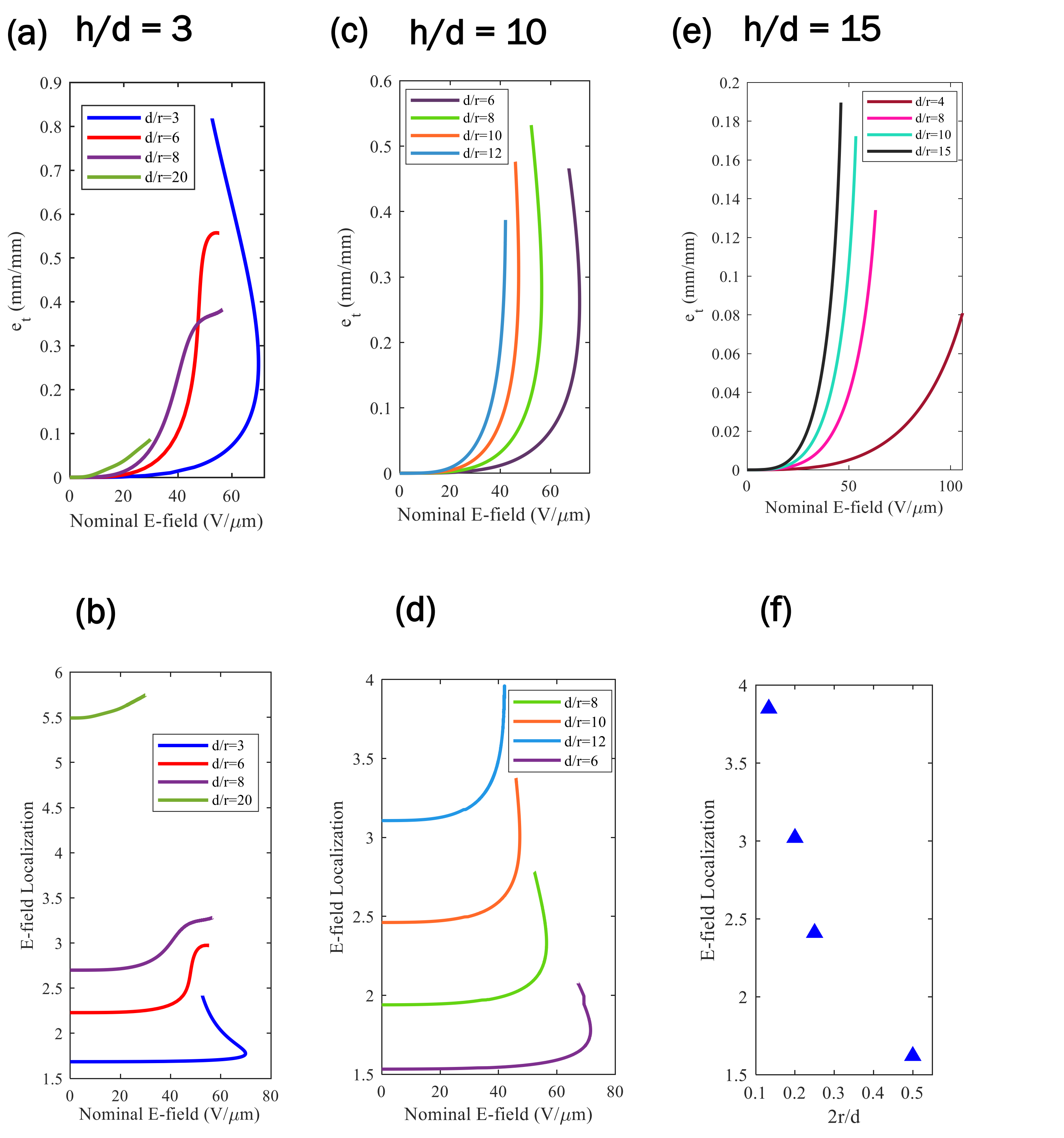

Figure S1: (a) Thickness actuation strain and (b) field localization (E_{max}/E_{avg}) as a function of d/r (h/d=3). (c) Thickness actuation strain and (d) field localization as a function of d/r (h/d=10). (e) Thickness actuation strain and (f) low-field localization as a function of d/r (h/d=15).

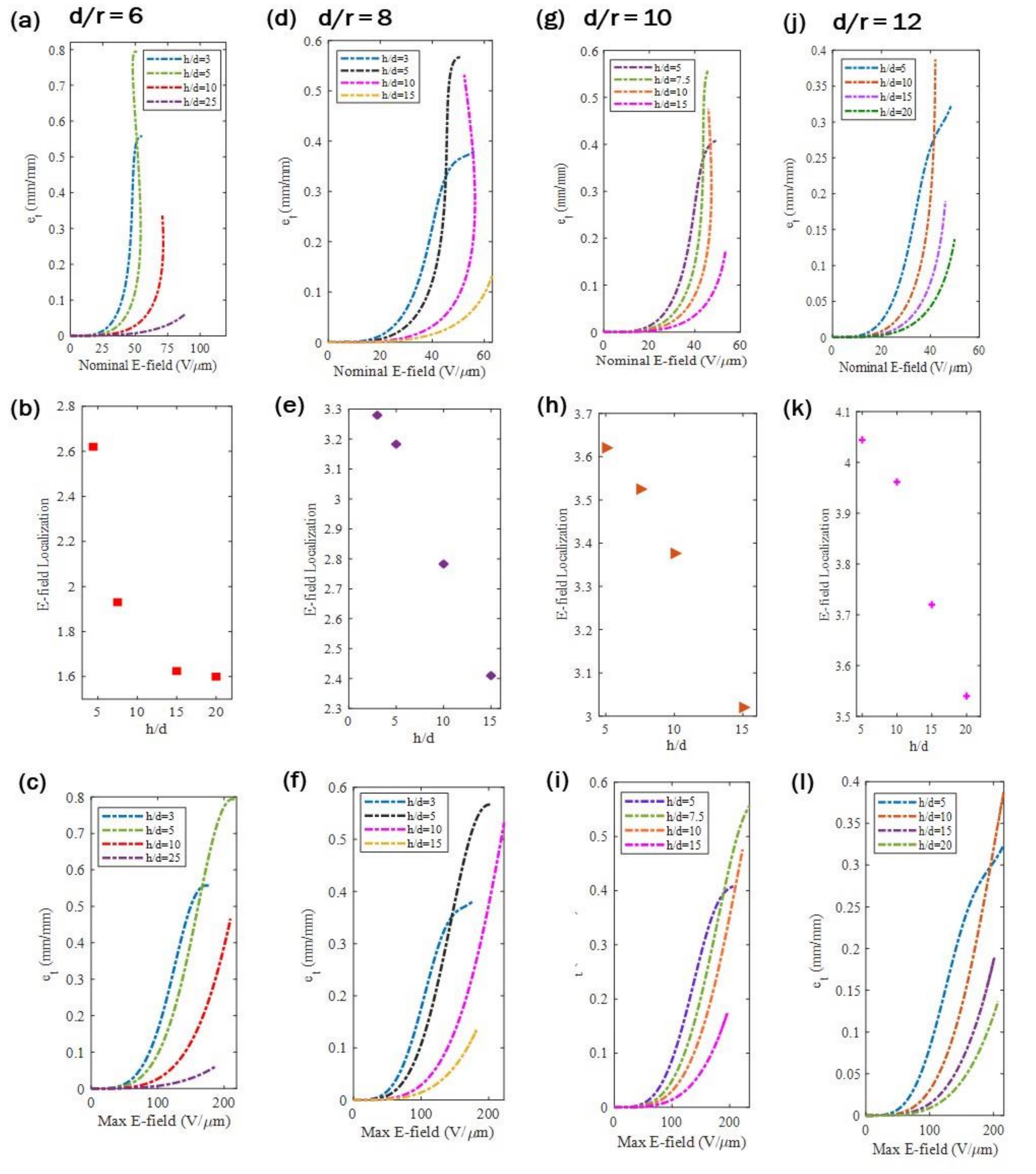

Figure S2: (a) Thickness actuation strain, (b) field localization (E_{max}/E_{avg}), and (c) strain vs max(E) as a function of h/d (d/r=4). (d) Thickness actuation strain, (e) field localization (E_{max}/E_{avg}), and (f) strain vs max(E) as a function of h/d (d/r=4). (g) Thickness actuation strain, (h) field localization (E_{max}/E_{avg}), and (i) strain vs max(E) as a function of h/d (d/r=4). (j) Thickness actuation strain, (k) field localization (E_{max}/E_{avg}), and (l) strain vs max(E) as a function of h/d (d/r=4).

Supplementary Note 2: Estimation of Sheet Resistance

One of the motivations for investigating the use of fiber-type electrodes is the potential for improved sheet resistance that could improve the actuation speed and reduce the heat generation during actuation. The theoretical sheet resistance is estimated by converting the cross-sectional area of the fiber electrodes to an equivalent electrode thickness. For a device with 2r/d=0.333 and h/d=3, the electrode radius (r) is h/18. The fiber area is $A = \pi r^2$ over a length of d. The equivalent thickness is then $t = \pi r^2/d$. The sheet resistance (R_s) is related to the resistivity through $R_s = \rho/t$. The resistivity of silver is $15.6 \cdot 10^{-9}$ Ω·m. Assuming a metal-based composite with a resistivity 10 times higher than silver, the resistivity of the fibers could be $156 \cdot 10^{-9}$ Ω·m. Consequently, the assumptions used in this analysis gives a sheet resistance of:

$$R_s = \frac{\rho d}{\pi r^2} = \frac{\rho(h/3)}{\pi(h/18)^2} = \frac{156 * 10^{-9} * (h/3)}{\pi(h/18)^2} \tag{19}$$

The resulting RC time constant is expected to be:

$$RC = 2 * (electrode\ resistance) * C$$
$$RC = 2 * \left(\frac{\rho L}{Wh}\right)\left(\frac{\epsilon_r \epsilon_0 WL}{h}\right) \tag{20}$$

where h is the thickness of the dielectric, W is the width, and L is the length. Assuming a square device with W=L,

$$RC = 2 * R_s * \left(\frac{\epsilon_r \epsilon_0 L^2}{h}\right) \tag{21}$$

Consequently, the expected RC time constant per square meter of dielectric can be approximated as:

$$\frac{RC}{L^2} = 2 * R_s * \left(\frac{\epsilon_r \epsilon_0}{h}\right) \tag{22}$$

For a device with a dielectric thickness (h) of 40 µm, the optimal fiber distribution (2r/d=0.333 and h/d=3) gives a fiber radius of 2.22 µm. Assuming the electrode material has a conductivity ~10% of bulk silver, the effective sheet resistance would be ~0.136 Ω/□.

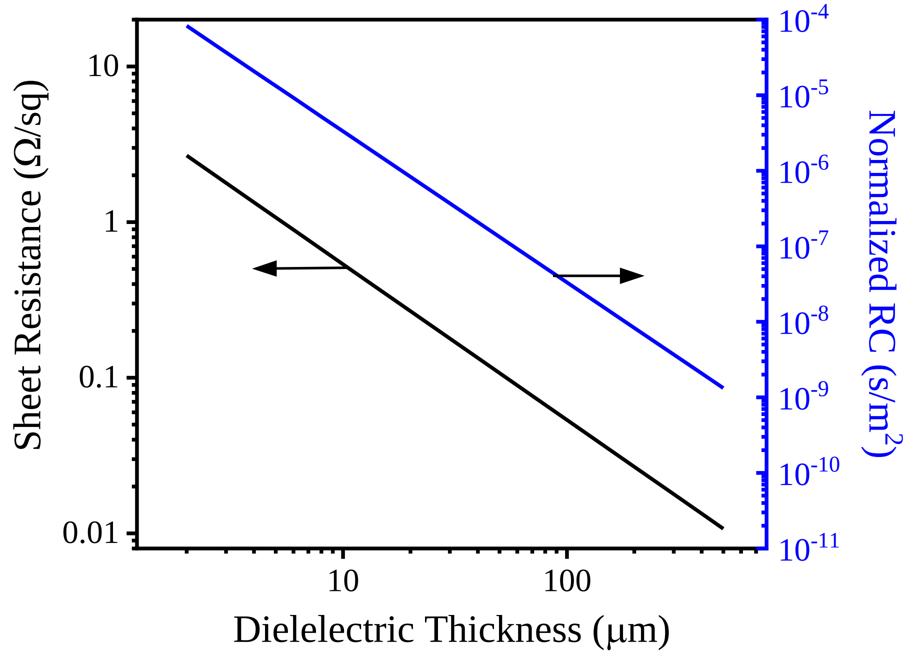

Figure S3: Predicted sheet resistance and RC time constant for AED devices.
